# Migraine, attention deficit hyperactivity disorder and screen time in children attending a Sri Lankan tertiary care facility: are they associated?

**DOI:** 10.1186/s12883-020-01855-5

**Published:** 2020-07-08

**Authors:** Udena Ruwindu Attygalle, Gemunu Hewawitharana, Champa Jayalakshmie Wijesinghe

**Affiliations:** 1 Teaching Hospital, Karapitiya, Galle, Sri Lanka; 2 Department of Community Medicine, Faculty of Medicine, University of Ruhuna, Galle, Sri Lanka

**Keywords:** Migraine, Attention deficit hyperactivity disorder, Screen-time, Children, Adolescents

## Abstract

**Background:**

Headache and Attention Deficit Hyperactivity Disorder (ADHD) are two relatively common, neuropsychiatric conditions seen in children. Recent studies have shown an association between these two disorders, which are otherwise distinct conditions. This study aims to assess the association between migraine and ADHD, as well as the association between screen-time and these two conditions, among children attending a Sri Lankan tertiary care facility. Possible associations will have important implications in the clinical management of these conditions.

**Methods:**

This was a comparative cross-sectional study of 226 children aged 5–14 years, attending clinics at a tertiary care hospital in Galle, Sri Lanka. Of them, 141 had a diagnosis of migraine and 85 did not have migraine. The presence or absence of ADHD and the use of screen-time among the two groups was analysed. Chi-square test and Mann-Whitney U test was used to assess the associations between these variables.

**Results:**

Approximately 5% of the children with migraine had clinically diagnosed ADHD, compared to 3.5% of those without migraine (*p* = 0.862). The median SNAP-IV scores (inter-quartile range) of the children with migraine and without migraine were 0.60 (0.27–1.00) and 0.44 (0.16–0.80) respectively (*p* = 0.014). There was no significant difference in screen-time hours per day between children with and without clinically diagnosed ADHD. However, a significant difference in median screen-time (hours per day) was observed between children with and without migraine (2.0 h and 1.0 h respectively; *p* = 0.012).

**Conclusions:**

Our findings suggest that children with migraine are more likely to show features of hyperactivity/impulsivity and inattentiveness than those without migraine. While no association was found between clinically diagnosed ADHD and screen-time, migraine was associated with longer daily screen use. Screening for ADHD in children diagnosed with migraine may be of benefit. Further studies are required to understand the possible benefits of reducing screen-time in children with migraine.

## Background

Headache is a common problem among children and adolescents, with 82% reporting at least one severe episode by 15 years of age [1]. Migraine meanwhile is described as the commonest disabling condition among headache disorders [2]. In the Global Burden of Disease Survey 2010, it was ranked as the third most prevalent disorder, and the seventh-highest cause of disability worldwide [3]. Attention Deficit Hyperactivity Disorder (ADHD) is another common condition in childhood. ADHD prevalence estimates are in the range of 2–12% in children and 1–4% in adults [4–8].

Migraine and ADHD are both conditions that have neurological and psychiatric dimensions [9, 10]. Both these conditions have been well researched over a long period of time. Although, it is only recently that researchers have begun to look for an association between the two, co-morbidity between migraine and several other psychiatric disorders has been well documented [9, 11–14]. Such co-morbidity is particularly seen in mood and anxiety disorders.

Compared to ADHD, migraine has a very different symptom profile and age and sex distribution. Migraine is an episodic disorder, with attacks of pain and time-limited neurological dysfunction [15]. Migraine has two major subtypes. Migraine without aura is a clinical syndrome characterized by headache with specific features and associated symptoms such as nausea, vomiting, photophobia, etc. Migraine with aura is primarily characterized by the transient focal neurological symptoms that usually precede or sometimes accompany the headache [16]. Other subtypes of migraine include hemiplegic migraine, abdominal migraine, basilar migraine, cyclical vomiting etc. Diagnosis of migraine is entirely clinical, but in some instances may need investigations including neuro imaging to exclude other causes such as in children with hemiplegic migraine [17]. Further brain imaging may be important to localize affected cerebral vascular territory [18].

In contrast, ADHD is a chronic disorder comprising of problems with attention and concentration, combined with behavioural symptoms such as hyperactivity and impulsivity. It is a disorder that can have a considerable effect on the day to day functions of those affected. It can impact academic performances, lead to rejection from school as well as dysfunction within families, due to the stress caused by the manifestations of this disorder. While predominantly contributed to by genetic factors, environmental factors can mitigate or exacerbate how symptoms present in this condition. While there is no common model to explain how both these disorders arise; a complex genetic aetiology has been postulated to be contributory in both conditions [19–21].

The impact of both these disorders on children can be severe, affecting academic work, school attendance, peer relationships and increased burden on families [4, 22, 23]. Early identification and treatment of these disorders when they are co-morbid, is likely to improve treatment outcomes.

Several recent studies have indicated a positive relationship between ADHD and migraine, in both children and adults [13, 24–29]. A recent meta-analysis reported a positive association between these two conditions [24]. A large community based study in Brazil showed that children aged 5–12 years with migraine were as seven times more likely as other children to have ADHD [26]. In contrast, there are also studies that have found ADHD to not be a significant risk factor for increased headache in children [30]. However, these findings have been in relation to headaches in general and not limited to migraine.

The use of technological devices and inherent increase in screen-time has been an area that has generated interest among researchers into both conditions. Screen-time related activities have been found to be associated with both conditions in the past [31–34], particularly in larger studies involving preschool children as well as young adults.

There are no major studies in South Asian populations on the association between migraine and ADHD. Data regarding screen-time in children is also scarce. Therefore, research into the association between these variables will provide information that will be useful in the management of both conditions, thus contributing to improving the quality of life of these children. As such, the objectives of the current study were to assess the association between migraine and ADHD among children attending a Sri Lankan tertiary care hospital and to evaluate the relationship of parent-reported screen-use with ADHD and migraine in these children.

## Methods

This comparative, cross-sectional study was conducted at the Teaching Hospital, Karapitiya in Galle, Sri Lanka, which is the major tertiary care institution in the Southern province of the country. The study was conducted from September 2018 to April 2019.

### Participants

Two groups of children aged 5–14 years, with and without migraine were recruited for the study using convenience sampling.

- A group of 141 children receiving treatment for migraine at the Paediatric Neurology Clinic were included in the ‘migraine group’. These children had already been diagnosed with migraine and most were on treatment which included medication. Children with co-morbid seizure disorders, intellectual disability/mental retardation and Autism Spectrum Disorders were excluded as these are more likely to be associated with ADHD.
- A group of 85 children without migraine who were attending the Outpatient Department (OPD) of the same hospital for minor health conditions or vaccinations were recruited as the ‘comparison group’. Those with intellectual disability/mental retardation, seizures, and Autism Spectrum Disorders as well as those presenting with headaches were excluded from this group as well.

The children in both groups were assessed by a Consultant Paediatric Neurologist to verify the presence or absence of migraine. The diagnosis of migraine was made according to the International Classification of Headache Disorders (ICHD-3) [35].

### Study instruments and data collection

A short, pre-tested questionnaire was used to collect data on basic socio-demographic characteristics of the children and daily and weekly screen time. Screen-time included the use of devices with screens, including phones, laptops and televisions. The data were collected from parents. Pre-testing of the questionnaire was done on a sample of 20 parents of children attending the OPD of the Mahamodara Hospital located nearby. The questionnaire was completed by the parents/caregivers.

To assess the presence of ADHD, the children in both groups were initially screened using a validated ADHD screening tool - the abbreviated version of the Swanson, Nolan, and Pelham (SNAP) Questionnaire (SNAP-IV), which has been successfully used in other studies involving Sri Lankan children [36, 37]. This parent/teacher rated tool assesses issues related to the domains of hyperactivity/impulsivity and inattention. A score of 1.67 or above was used to identify children who are likely to have ADHD, based on the 5% cut-off threshold for ADHD (Combined) according to parent rating as suggested in the scoring instructions. This was also the cut off value used in screening children for ADHD in routine clinical practice in Sri Lanka, with a sensitivity of 91% and a specificity of 88% as reported in local literature [36]. The children with positive scores and marginal scores on the screening tool were subjected to a confirmatory clinical interview conducted by a Consultant Child and Adolescent Psychiatrist using the DSM-5 (Diagnostic and Statistical Manual of Mental Disorders version 5) criteria for diagnosis to verify the diagnosis of ADHD. Information from other sources such as school was gathered where needed.

The participants were recruited after obtaining written informed consent from the parents as well as the assent of the children. Prior administrative approval for data collection was obtained from the relevant hospital authorities. The data collection was done by the research team after an initial training, and all clinical assessments were conducted by the same clinicians to minimize inter-observer variation. Confidentiality of the information and the anonymity of the subjects was strictly maintained and the children diagnosed with ADHD were offered treatment at the Child Psychiatry clinic.

### Data analysis

The statistical program SPSS (version 20.0) was used for data analysis. Descriptive statistics such as means/medians and proportions were calculated. Comparisons were made between children with and without migraine, with regards to the presence of ADHD and screen-time. In addition, the possible association between ADHD and screen-time was also assessed. In the absence of a consensus on recommended daily screen time for children over 5 years of age, a cut off threshold of over 1 h of screen-time was considered as a longer screen time. Chi square test or Mann-Whitney U test was used to determine the statistical significance of the observed associations.

## Results

There were 226 participants in total. Of them 112 (50.2%) were male and 111 (49.8%) were female. Three participants had not reported their sex. Data on age was available in 224 participants, with a mean age of 10 years and a standard deviation (SD) of 2.7 years.

There were 141 participants who were identified as having migraine (mean age ± SD = 10.6 ± 2.4 years; 56.8% females) and 85 without migraine (mean age ± SD = 9.2 ± 2.8 years; 38.1% females); the differences in age and sex were statistically significant (*p* < 0.01). There were 10 children (4.4%) who fulfilled criteria for ADHD and 216 (95.6%) without ADHD based on clinical diagnosis, which was almost similar to the numbers detected during initial screening using SNAP-IV; *n* = 11 (4.9%) and *n* = 215 (95.1%), respectively. None of these children had a previous diagnosis of ADHD or any form of unaddressed issues related to functioning.

### Association between migraine and ADHD

Of the children with migraine, approximately 5.0% (*n* = 7) had clinically diagnosed ADHD. In comparison 3.5% (*n* = 3) of children without migraine had ADHD, though this difference was not statistically significant (*p* = 0.862). However, a statistically significant difference was observed between the median SNAP-IV scores of the children with migraine and those without migraine (*p* = 0.014), showing a higher median score among children with migraine (Table 1).

**Table 1:** Distribution of SNAP-IV scores in children with and without migraine in the sample

Presence of migraine	N	SNAP-IV scores		Significance
		Mean (SD)	Median (IQR)	
Yes	141	0.69 (0.53)	0.60 (0.27–1.00)	U = 4825.000 *p* = 0.014*
No	85	0.53 (0.49)	0.44 (0.16–0.80)	

^*^Differences in median were statistically significant at 0.05 level; Mann-Whitney U test

The children with both migraine and ADHD were found to have a significantly higher proportion of females (60.0%), compared to those without any disorder (37%) or with only one of the two conditions (56.8%; *p* = 0.016). In contrast, no significant differences were observed in age distribution of the three categories, though the mean age of the group with co-morbidity was found to be lower than the rest of the sample (8.7 and 10.1 years respectively; *p* = 0.115).

### Association between migraine, ADHD and daily screen-time

Screen-time data were available for 113 participants. Of them, 107 (94.7%) reported regular daily screen-time and six participants (5.3%) reported no allowance of daily screen-time. The mean ± SD daily screen-time of the sample was 1.9 ± 1.2 h. The median daily screen-time (IQR) was two (1–3) hours. The distribution of daily screen-time of children according to migraine and ADHD status are shown in Table 2.

**Table 2:** Distribution of daily screen-use time according to migraine and ADHD status of the children in the sample

Presence/Absence of the condition	N	Daily screen-use time (hours per day)		Significance
		Mean (SD)	Median (IQR)	
**ADHD**				
Present	4	3.0 (2.7)	2.0 (1.25–5.75)	U = 161.000 *p* = 0.449
Absent	103	1.89 (1.13)	2.0 (1.0–3.0)	
**Migraine**				
Present	40	2.29 (1.25)	2.0 (1.13–3.0)	U = 959.500 *p* = 0.012*
Absent	67	1.72 (1.16)	1.0 (1.0–2.0)	

^*^Differences in median were statistically significant at 0.05 level; Mann-Whitney U test

The results show that the median screen-time (hours per day) was not significantly different between the children with and without clinically diagnosed ADHD. However, a significant difference in the median screen-time hours per day was seen between children with and without migraine. The children with migraine on average reported a higher duration of screen-time per day (*p* < 0.05) (Table 2).

To further analyse this association, the daily screen-time use was categorized into two groups based on a cut off threshold of over 1 h, which was considered as a prolonged screen-time. Of those with details of daily screen use time, 62 (57.9%) reported a daily screen-time of over 1 h. Association of migraine and ADHD status with a prolonged screen-time is shown in Table 3.

**Table 3:** Distribution of prolonged screen-time according to migraine and ADHD status of the children in the sample

Presence/Absence of the condition	Daily screen-use time				Total		***p*** value
	≤ one hour		> one hour				
	No	%	No	%	No	%	
**ADHD**							
Present	1	25.0	3	75.0	4	100.0	0.851
Absent	44	42.7	59	57.3	103	100.0	
**Migraine**							
Present	10	25.0	30	75.0	40	100.0	0.006*
Absent	35	52.2	32	47.8	67	100.0	

^*^Differences were statistically significant at 0.001 level; Chi square test

The findings confirmed the association observed in Table 2, indicating that a significantly higher proportion of children with migraine reported having daily screen-times of more than 1 h, compared to those without migraine (75.0% Vs. 47.8%; *p* < 0.01). In contrast, the children with or without clinically diagnosed ADHD failed to show such an association (Table 3).

## Discussion

This study assessed the association between migraine, ADHD and screen-time in a group of Sri Lankan children attending a tertiary care hospital. To our knowledge, a study of a similar nature linking these variables has not been conducted in Sri Lanka or in the Asian context previously.

The findings of this study points to higher levels of perceived hyperactivity/impulsivity and inattention among children with migraine (Table 1). However, this did not translate into an association with clinically diagnosed ADHD that was statistically significant. Previous studies have pointed to an association between these two disorders using various study instruments [24–27]. This association is also been reported across child and adolescent [24–26] as well as adult populations [27]. While the mechanism for the association is unknown, these studies did not find the same association with tension type headaches [24, 26]. As tension type headaches are more related to environmental stressors this points to the association being more biological than environmental.

Interestingly, ADHD is a disorder more commonly found in males [38] and migraine is more commonly found in females [22]. Meanwhile, some studies on adult populations have found that this co-morbidity increases with age and was more common in females [27]. In the current study we found that a significantly higher proportion of children with migraine were females and they had a higher mean age, compared to those without migraine in keeping with the existing findings. However, with regards to co-morbidity, our findings confirmed only the reported association with gender and a contrasting non-significant difference was observed with age.

Previous studies on child and adolescent populations have found that the association with migraine was limited to the hyperactivity impulsivity domain of ADHD and not the inattention domain [26]. Our findings however, showed an association with overall ADHD scores, indicating that attention was affected as well. Similarly other studies do not describe specific domains when discussing this association [39]. Although the scores were such, other than those diagnosed with ADHD, the others in the migraine group did not report major functional issue due to hyperactivity or inattention.

There have also been studies on the association between migraine and other psychiatric disorders such as Bipolar Affective Disorder [11]. In the case of bipolar disorder it has been suggested that when it is associated with migraine, it might represent a more severe variant of the disorder. However, the literature is unclear on greater morbidity when ADHD is associated with migraine [24–27].

Meanwhile, ADHD symptoms and screen use, or internet/gaming addiction has been thought to share a bidirectional relationship. These activities are postulated to be more attractive, to those with inattention/impulsivity issues and in turn screen-use may exacerbate inattention/impulsivity symptoms by providing an activity that continuously reinforces disinhibition, quick responsiveness and need for immediate reward [31]. This model however remains to be clearly established. Meanwhile the current study did not show an association between daily screen-time and ADHD (Tables 2 & 3). In contrast, previous studies in large samples have shown an association between parent-reported screen-time and inattention problems in Canadian preschool children [33] as well as screen-time and self-reported inattention hyperactivity in French university and higher education students [34]. The contrasting findings in present study may have been contributed to by the small numbers of children with ADHD in the sample. Further studies are recommended using a lager sample of children with ADHD to establish whether there is an association between the two conditions.

On the contrary, there was a significant association between daily screen-time and migraine in children (Tables 2 & 3), which is comparable with available limited adult based studies [32]. These findings suggest that further research is needed to clarify the effects of screen use in children with migraine.

While this study is limited by the small sample size, it adds useful knowledge to the existing evidence base, particularly in an Asian context. Drawing a sample from children attending a tertiary care hospital where there would be a higher representation of children with more severe or complicated conditions may affect the generalizability of findings. It is also possible that as the screen-time was parent-reported, it may differ from actual screen-time. Parents may not be aware of some of the times that their children spend with screens.

Further, certain confounding factors that can lead to presentations of hyperactivity and inattention similar to ADHD such as obstructive sleep apnoea syndrome, insomnia and obesity were not excluded upon subject recruitment. However, its effect would have been negligible as all the children who were screened positive for ADHD have undergone a clinical assessment to confirm the diagnosis of ADHD.

The conclusions of the study were also limited by the available evidence with regards to what is an acceptable level of screen-use. The physical activity recommendations for children less than 5 years published by the World Health Organization states to limit sedentary screen time for no more than 1 h [40], however, no such threshold is suggested for older children. Thus, our findings only show a comparative increase in daily screen use in children with migraine. There may be many other social and cultural factors that may have contributed to this difference. As most of the children with migraine were already on medication this too may have had an effect on the results.

## Conclusion

Our findings suggest that children with migraine are more likely to be hyperactive/impulsive and inattentive than those without migraine. While no association was found between ADHD and screen-time, migraine was associated with longer daily screen use. Screening for ADHD in children diagnosed with migraine may be of benefit. Further studies are required to understand the possible benefits of reducing screen-time in children with migraine.

## Data Availability

The data sets used and/or analysed during the current study are available from the corresponding author on reasonable request.

## Abbreviations

ADHD: Attention Deficit Hyperactivity Disorder
DSM: Diagnostic and Statistical Manual
ICHD: International Classification of Headache Disorders
IQR: Inter-quartile Range
OPD: Out Patient Department
SD: Standard Deviation
SNAP-IN: Swanson, Nolan and Pelham-IV
SPSS: Statistical Package for Social Sciences
